# Creatine monohydrate in ALS: Effects on strength, fatigue, respiratory status and ALSFRS

**DOI:** 10.1080/17482960802028890

**Published:** 2009-07-10

**Authors:** Jeffrey Rosenfeld, Ruth M. King, Carlayne E. Jackson, Richard S. Bedlack, Richard J. Barohn, Arthur Dick, Lawrence H. Phillips, John Chapin, Deborah F. Gelinas, Jau-Shin Lou

**Affiliations:** ^a^ The Carolinas Neuromuscular/ALS Center, Charlotte North Carolina, Carolinas Medical Center; ^b^ University of Texas Health Science Center, , San Antonio, Texas; ^c^ Duke University, Durham, North Carolina; ^d^ University of Kansas, Kansas City; ^e^ University of Virginia, Charlottesville, Virginia; ^f^ University of New Mexico, Albuquerque, New Mexico; ^g^ California Pacific Medical Center, San Francisco, California; ^h^ University of Oregon, , Portland Oregon, , USA

**Keywords:** Creatine, amyotrophic lateral sclerosis, strength, fatigue

## Abstract

Our objective was to determine the effect of creatine monohydrate on disease progression in patients with amyotrophic lateral sclerosis (ALS). One hundred and seven patients with the diagnosis of probable or definite ALS, of less than five years duration from symptom onset, were randomized to either treatment with daily creatine monohydrate (5 g/d) or placebo. In this multicenter, double-blinded study we followed changes in disease progression: using quantitative measures of strength via maximal isometric voluntary contraction, forced vital capacity, ALSFRS, quality of life, fatigue and survival. Patients were followed for nine months. The results showed that creatine monohydrate did not significantly improve motor, respiratory or functional capacity in this patient population. The drug was well tolerated and the study groups well balanced, especially considering the absence of forced vital capacity criteria for entrance into the study. There was a trend toward improved survival in patients taking daily creatine monohydrate and this was identical to the trend seen in another recently published report of creatine in ALS patients [Bibr CIT0001]. In conclusion, creatine monohydrate (5 g/d) did not have an obvious benefit on the multiple markers of disease progression measured over nine months. We measured fatigue during isometric contraction and found no significant improvement despite anecdotal patient reports prior to and during the study. The trend toward improved survival was also found in another recently completed blinded trial using creatine monohydrate. Further investigation on the possible survival benefit of creatine in this patient population is ongoing.

## Introduction

The mechanism of amyotrophic lateral sclerosis (ALS) involves a complex inter-relationship of various biochemical processes. Energy metabolism, glutamate homeostasis, calcium metabolism and the immune system can all contribute [2]. Mitochondrial dysfunction has been implicated as a pivotal component in several parallel pathways [3]. Attempts to enhance mitochondrial function have, therefore, generated significant interest as potential therapy for patients with ALS.

Creatine is a naturally occurring guanidine-derived compound that is synthesized endogenously in humans in the liver and is found predominantly in muscle and brain [4–6]. The enzyme creatine kinase catalyzes the phosphorylation of creatine and the dephosphorylation of phosphocreatine.

The daily requirement for creatine supplied through the diet or from endogenous synthesis is approximately 2 g per day [7]. For most individuals, the diet provides an estimated intake of up to 1 g per day [8]. Supplemental creatine may increase maximal availability for energy output in anaerobic activities [9–11] and can also stimulate mitochondrial respiration and phosphocreatine synthesis [12]. These mechanisms may help explain the apparent neuroprotective properties of creatine seen in a transgenic model of ALS, as well as anecdotal reports of increased energy in patients with ALS [13]. Beneficial effects of creatine on muscle fatigue have been demonstrated in patients with a variety of muscular dystrophies [14], [15].

Creatine has been systematically studied in humans with ALS on at least three occasions. Prior to the onset of this study, a nine-month placebo-controlled, pilot study of creatine monohydrate in 21 patients with ALS showed either a significantly greater improvement in strength as measured by maximal voluntary isometric contraction (MVIC) or a more modest decline compared to patients taking placebo [16]. The most profound effects were noted at the earliest time point(s), within the first month. The pilot study also showed a trend favoring an improvement in FVC associated with improved MVIC in the creatine group. The conclusions from these data were, however, limited due to the relatively small number of patients in the pilot study. Based on these data, the current multicenter, placebo-controlled study was initiated.

Two larger randomized trials using creatine in the ALS population have also been completed [1], [17]. Both trials, differing in design and endpoints, failed to demonstrate a significant treatment effect. The current trial was designed to examine the rate of change (slope) of motor function in individual muscle groups as well as the fatigability of muscle strength, indices not fully explored previously. Emphasis was placed on the earliest time points where the most profound treatment effect was noted in the pilot study.

## Methods

### General study design

The study was designed as a double-blind, placebo-controlled trial conducted at nine sites within the USA. All sites obtained local institutional review board (IRB) approval prior to initiating the study. Patients were randomized in a 1:1 ratio to receive either ultra-pure creatine monohydrate or placebo. Patients received either 10 g of creatine daily for five days followed by 5 g daily for the remainder of the study or matching placebo. Creatine powder and matching placebo were provided by the Avicena Group Inc. Randomization was organized in blocks and stratified by clinic to ensure treatment group balance at each site. All serious adverse events were reported to an independent Data Safety Monitoring Board (DSMB) twice yearly. The DSMB had the ability to recommend changes to the study protocol or discontinue the study due to safety concerns.

### Patients

Eligible subjects had a diagnosis of laboratory-supported, probable or definite ALS according to El Escorial criteria [18], were between the ages of 21 and 80 years, and had a disease duration of less than five years from symptom onset. Subjects had at least five of 10 testable upper extremity muscle groups of MRC grade 4 or better, and, if receiving riluzole, the only FDA approved treatment for ALS, they were on a stable dose for at least 30 days. Women of childbearing potential were non-lactating and surgically sterile or using an effective method of birth control and had a negative pregnancy test prior to randomization.

Forced vital capacity was not used as an eligibility criterion; however, subjects were not eligible if they required tracheostomy ventilation, had a diagnosis of other neurodegenerative diseases, renal disease, or clinically significant history of unstable medical illness over the previous 30 days. Subjects with a history of recent alcohol or drug abuse or non-compliance were excluded as well as subjects with limited mental capacity that prevented them from giving informed consent. Subjects that used creatine within 30 days prior to study initiation were also excluded.

### Study procedures

Prior to subject enrollment, each site demonstrated MVIC reliability by testing four normal subjects in duplicate with an intra-rater variability of less than 15% and an inter-rater variability of less than 20%.

Potential subjects were initially evaluated in a screening visit during which they signed an informed consent form and were assessed for eligibility. A medical history was completed and a physical examination was performed. Blood was collected to access baseline values for subsequent safety monitoring (BUN, creatinine, and urinalysis). In addition, patients collected a 24-h urine sample to assess baseline creatine levels. Eligible patients received baseline assessments including: maximal voluntary isometric contraction (MVIC), forced vital capacity (FVC), ALS functional rating scale revised (ALSFRS-R), the quality of life questionnaire short form 12 (SF-12), and muscle fatigue. Subjects were seen weekly for three weeks after randomization to assess acute benefits of creatine. Subjects then returned at months 2, 3, 4, 5, 7 and 9. The primary outcomes measurement, MVIC, was measured in 10 upper extremity muscles (shoulder flexion/extension, elbow flexion/extension and grip) at each visit. MVIC was performed with one 30-s trial for each muscle. MVIC scores were standardized and expressed as a percent of predicted values based on the subject's age, sex, height and weight [19], [20].

Secondary outcomes measures were evaluated throughout the study and included FVC, ALSFRS-R, SF-12 and muscle fatigue. FVC was calculated based on the best of three trials and standardized by percent predicted calculations. Muscle fatigue was determined by the subject's ability to sustain maximal voluntary isometric contraction. The area under each subject's force versus time curve was compared to the expected area under the curve in the absence of fatigue. This ratio provided a fatigue index (FI) [20] for each patient.

### Statistical analysis

The sample size was estimated using the variance formula for slope that was provided in Diggle et al. [21]. It adjusts the variance of the outcome measure by the correlation among the repeated measurements within a subject over time. This formula involves the standard deviation of the response variable, correlation between measurements at two different times within subject (assumed to be constant across subjects and between times). Estimates of these parameters were obtained from the pilot study of creatine versus placebo performed by the Carolinas Neuromuscular/ALS Center and the University of Texas Health Science Center at San Antonio prior to the initiation of the current study. Sample size was corrected by the factor 1/(1 − d) = 1/(1−.10) = 1.1 [22]. The required total sample size to detect a 15% decrease in the rate of change of a composite MVIC score with 85% power is 94 (47 per treatment group).

To meet this sample size estimate 107 subjects were randomized, 53 in the creatine group and 54 in the placebo group. Thirty patients did not complete the study (13 in the creatine group and 17 in the placebo group) including eight patients who died during the study (two in the creatine group and six in the placebo group).

### Analysis

The intent-to-treat (ITT) principle was adopted for all analysis. Thus all randomized subjects, regardless of whether they completed the study per protocol, were included in the analysis. Missing values for individual study visits were excluded from determinants of slope for those patients. In patients who did not complete the study or those who died prior to study completion, only actual collected data values were used in the analyses, i.e. no data values were imputed. The primary efficacy outcome measure, muscle strength via MVIC was analyzed using a mixed model analysis of variance (random coefficient model) [21], [23]. This method is analogous to a comparison of the rate of change (or the slope of the linear time trend) of the outcome measure between treatment groups.

The model contained three fixed effect terms – treatment group, time, treatment group by time interaction – with subject and subject by time interaction [23] as random effects. The Type III F-statistic corresponding to the treatment group by time interaction term tests the hypothesis of the common slope between the treatment and control groups.

Muscle fatigue was analyzed using repeat measures analysis of variance to compare changes in the scores over time and between muscle groups as well as for the overall average score of all 10 muscles. Repeat analysis of variance and the hierarchical linear model (HLM) procedures were used to analyze the rate of change in FVC. For ordinal data (ALSFRS-R, SF-12) the change in score from baseline to nine months was compared between the two groups with a Wilcoxon rank-sum test. For each of the groups separately, Friedman's test was used to test for changes over time. A multivariate analysis of variance (MANOVA) was employed to compare the subscales of the SF-12 between the groups. The total number of adverse events as well as abnormal laboratory results were compared between the two groups using Fisher's exact test. The number of subjects who completed the study in both groups was also compared using Fisher's exact test.

The two groups were compared for differences in demographic and baseline measures. Pairwise comparisons (*t*-test) were used for data measured on the interval scale (age, MVIC, FVC), Wilcoxon rank sum tests for ordinal data (ALSFRS-R, SF-12), and χ^2^ tests for nominal data (gender). The Shapiro-Wilk test was used to test interval data for normality. If the data were not normally distributed, the appropriate non-parametric test was employed. A *p*-value of less than 0.05 was considered statistically significant.

## Results

### Study population

One hundred and seven patients were enrolled in the trial including 62 males and 45 females (*p* >0.9). These patients were randomized 1:1 to receive creatine (*n = *53) and placebo (*n = *54). Baseline demographics and functional indices are shown in Table I. The study was statistically balanced between treatment and placebo groups.

**Table I.** Baseline values for enrolled patients.

	Creatine	Placebo
*n*=	53	54
Gender	35 males, 18 females	27 males, 27 females
Age	56±10	59 ±11
ALSFRS	36±6	36±7
FVC	76.2%	78.2%
Symptom onset	1.5 years	1.9 years
Limb:Bulbar onset	2.7:1	4.4:1

### Strength measurements

**Figure 1.**
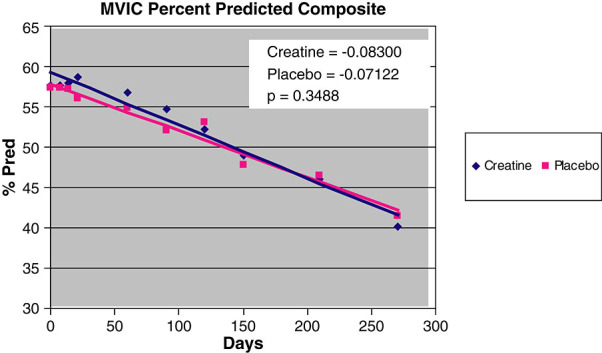
Slope of decline in MVIC in patients taking creatine and those taking placebo over the nine-month study period.

The decline in MVIC was not statistically different in the patients receiving creatine (−0.08±0.01) and placebo (−0.07±0.01) treatments over the nine-month study period (Figure 1). Similarly, the slope of declining muscle strength was stratified to examine whether there might be a selective benefit to either stronger or weaker muscle groups (three groups: <40%, 40–69%, ≥70%). No significant differences were found for muscle groups that were either stronger or weaker at baseline testing.

Individual muscle groups were also analyzed for selective effects using a time-to-failure analysis examining the time involved in two successive 10% declines in percent predicted MVIC. Significant differences were not found in this stratified analysis; however, a consistent trend in preserved strength was seen in muscles which were >70% of their predicted values at baseline. This trend did not reach statistical significance.

### Fatigue measurements

Similar analyses to those performed for MVIC were completed on measurements of muscle fatigue for each muscle group over the nine-month study. The slope in declining fatigue indices was not significantly different for patients taking placebo and creatine in any of the muscle groups tested.

Intrinsic hand muscles and forearm muscles contributing to grip strength were isolated and compared with larger proximal flexor and extensor muscles of the shoulder. No difference in the fatigability of weaker distal muscles compared with proximal muscles was noted within the treatment or placebo groups. Similarly, muscles stratified for baseline strength did not differ in their fatigue measurements.

### Forced vital capacity

Maximal forced vital capacity measurements were not significantly different between patients taking placebo and creatine at any of the time points measured in the study. Similarly, the percent predicted values for these measurements were not different between the two groups. Slope of decline in FVC was compared using the mixed model analysis of variance, across the nine-month study period, and no differences were found (*p = *0.3).

### SF-12 quality of life index

Observed decline in the SF-12 measure was not significantly different between the two treatment groups (*p = *0.7). Available subscores of the SF-12 (i.e. ‘physical’ and ‘mental’) were also not significantly different between groups at all time points tested.

### ALSFRS-R

No differences were found between creatine and placebo groups in measures of total ALSFRS-R at all time points sampled.

### Survival

**Figure 2.**
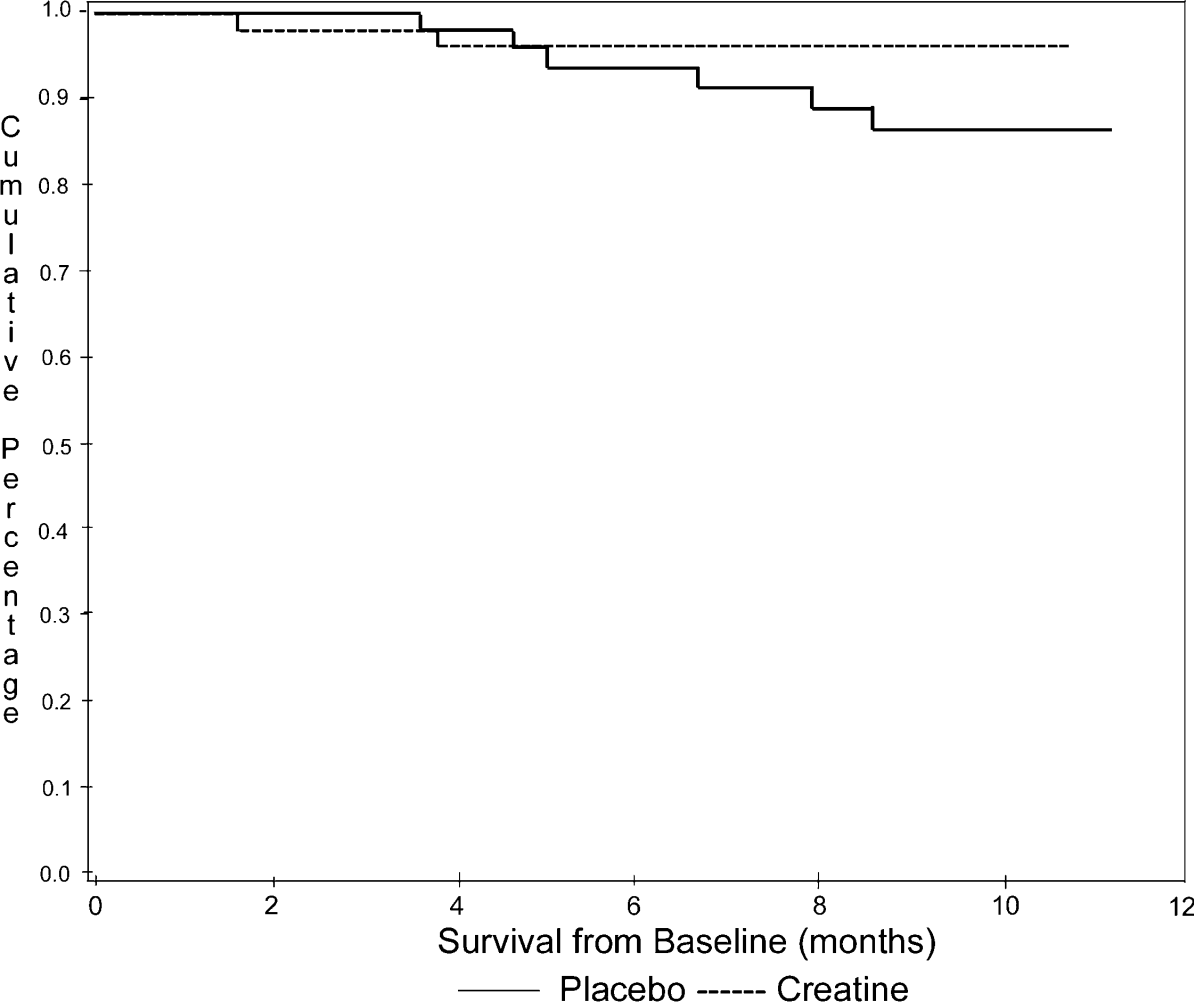
Survival.

Eight patients (7%) died during the study trial period. Two of these patients were in the creatine group and six were assigned to the placebo group. Although the study was not powered to examine a survival benefit, these data were tested in a log-rank analysis and the difference did not reach statistical significance (Figure 2).

### Compliance and safety

**Figure 3.**
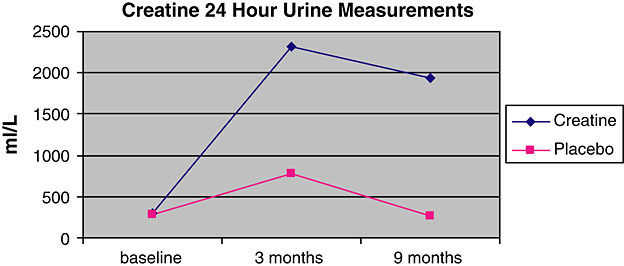
Compliance testing via 24-h urine-creatine measures.Creatine was measured in 24-hour urine collections at baseline, three and nine months. The creatine treatment group had a significant increase in measured creatine, as expected, compared with the placebo group. This indicator of compliance with assigned treatment group revealed significant compliance to the protocol, despite the availability of creatine in the community (see text). Creatine vs. placebo at baseline, *p*=0.367 Creatine vs. placebo at three months, *p*<0.00001 Creatine vs. placebo at nine months, *p*<0.00001

The creatine administered in the trial was well tolerated. No consistent adverse events were reported, and this may reflect the relatively low dose used compared with subsequent studies that used much higher doses (Table II). Creatine excreted in a 24-h urine sample was measured to estimate patient compliance with the treatment protocol (Figure 3). Patients assigned to the creatine treatment group had average values of excreted creatine six to nine times higher than in the placebo group or compared with baseline values at the onset of study. One patient in the treatment group had values of excreted creatine in the placebo range at three months. Only three patients in the placebo group had values consistent with those in the creatine group that indicated non-compliance. Overall the data contributed from these patients were not sufficient to result in a substantial effect in the data analysis.

**Table II.** Adverse events reported by patients on creatine and placebo. No statistically significant differences were found for any of the reported symptoms.

Adverse events	Creatine	Placebo
Falls and related injuries	25	39
Cramps	7	13
Diarrhea	6	12
Constipation	9	8
Back pain	9	7
Sinusitis	7	9
Headache not specified	4	11
Nasopharyngitis	5	9
UTI	4	10
Urinary frequency/urgency	6	4
PEG & PEG related AEs	5	7
Dyspnea	4	7
Nausea	2	7
Pneumonia	3	5
Respiratory failure	1	7
Bronchitis	4	3
Depression	3	4
Edema	3	4

## Discussion

The current study failed to demonstrate a therapeutic benefit of highly purified creatine monohydrate in tests of muscle strength, fatigability, forced vital capacity, ALSFRS-R and SF-12, in a large population of patients with ALS. This is the first study to examine muscle fatigability as an outcome from creatine monohydrate in ALS patients. Often, patients’ subjective reports include a perceived benefit in muscle fatigue and endurance while they are using creatine. We were unable to demonstrate such a therapeutic benefit. Despite the absence of significant efficacy, the fatigue testing procedures used here were well tolerated and could suggest an important alternative endpoint for future trials in this patient population.

Other measures of functional decline such as FVC, ALSFRS-R and SF-12 were similarly unaffected by the use of creatine in this trial. While the study was not powered specifically for these endpoints, this effect is consistent with conclusions of earlier trials of creatine in this patient population.

Our inability to demonstrate a significant clinical effect despite multiple anecdotal reports and significant data from our phase II trial may be due to several issues. Disease heterogeneity, higher creatine dosing and the sensitivity of our endpoints may be areas of further investigation in the future. Creatine may also have a selective benefit in individual patients with motor neuron disease given our current uncertainty of the significance of the marked variability observed in patients presenting with ALS. Our attempts to stratify patients with rapid or slowly progressive weakness were not sufficient to reveal selective effects. Future attempts to stratify patients based on the extent of lower versus upper motor neuron involvement, bulbar versus limb involvement, or slowly progressive disease versus fast progressing disease may be of potential benefit in identifying a selective benefit in a subgroup. Analysis of changes in slope of declining muscle strength, as performed in this study, seem most consistent with the disease course. Despite this, however, we were unable to detect a change in declining slope using repeat measures analysis of variance and the HLM procedures. Additional subgroup analyses, using stratified data, appropriately powered to detect selective effects in stronger versus weaker muscles at baseline may ultimately be more sensitive.

Eight patients died prior to the end of the trial. Six of these patients were in the placebo group and 2 in the creatine group. These data, while not statistically significant, generated an additional analysis that used survival data combined with a similar observation from another recently completed creatine trial.

Creatine monohydrate is readily available without prescription. Despite this availability, compliance with the treatment protocol was quite high. We detected only three patients in the placebo group (5%) who may have obtained creatine elsewhere; however, these data did not impact the overall analyses. This observation has implications for patient compliance in future trials of readily available compounds where a prori concern may be raised for adherence to the protocol.

The current trial did not use inclusion criteria for forced vital capacity. This most common entry criterion is often used to ensure patient longevity throughout the study period. Despite the absence of this screening tool and the presence of other concurrent trials that competed for patient enrollment, the study population was not adversely affected, and baseline demographics were not skewed toward patients with more advanced disease. This is an argument that is used frequently to justify FVC entry criteria. Our data suggest that rigorous FVC entry criteria into research trials may not be essential depending upon efficacy measures used in future trials.

Overall, creatine monohydrate was not found to have a significant clinical benefit in measures of fatigue, strength or respiratory capacity as tested in this trial. Recently completed trials in Huntington's disease (unpublished) suggest a much higher dose of creatine is safe and tolerable, and may be necessary for optimal benefit. Stratification of future data with sufficient patient numbers may also be necessary to explore whether there are subgroup(s) of ALS patients that may respond.

## Acknowledgements

The authors express their appreciation for the assistance in experimental design offered by Barbara Tilley (Medical University of South Carolina) and to James Norton (Carolinas Medical Center) for aid in the statistical analyses. We are especially grateful to the numerous study coordinators and research staff at the participating institutions listed below: Cathleen Stenger, Amy Swartz, Amber Ward (Carolinas Medical Center); Pam Kittrell, Deborah Meyers (University of Texas Health Science Center, San Antonio); Laura Herbelin (University of Kansas); Karen Grace, Young Choi (Duke University); Melanie Davis, Kevin Blaine (University of Oregon Health Science Center); Joan Warner, Martha Meister (University of New Mexico); Giovanna Kushur, Jason Mass (California Pacific Medical Center); Cindy Barnhill Fischer, Melissa Grove (University of Virginia).

This project was supported by the National Institutes of Health (NCCAM) RO1-AT00967-01. The Avicena Group generously supplied the study drug and placebo used throughout the trial. In addition, funding for the clinical research organization monitoring data entry was supplied by Avicena. This work was also supported by the National Center for Research Resources for the Frederic C. Bartter General Clinical Research Center.

Dr. Rosenfeld has served as a consultant for the Avicena Group, at the onset and early stages of this trial, for issues pertaining to the potential application of creatine in neurodegenerative disease.
